# Cerebrovascular alterations in a mouse model of late-onset Alzheimer’s disease

**DOI:** 10.1117/1.NPh.12.S1.S14614

**Published:** 2025-06-05

**Authors:** Christian Crouzet, Danny F. Xie, Maiella Nona Laquindanum Nuqui, Jonathan Hasselman, Thinh Phan, Robert H. Wilson, David Baglietto-Vargas, Celia Da Cunha, Hayk Davtyan, Stefania Forner, Amandine Jullienne, Afsheen Bazrafkan, Frank M. LaFerla, Andre Obenaus, Mathew Blurton-Jones, Yama Akbari, Kim N. Green, Bernard Choi

**Affiliations:** a University of California, Irvine, Beckman Laser Institute and Medical Clinic, Irvine, California, United States; b University of California, Irvine, Department of Biomedical Engineering, Irvine, California, United States; c University of California, Irvine, School of Information and Computer Science, Irvine, California, United States; d University of California, Irvine, Institute for Memory Impairments and Neurological Disorders (UCI MIND), Irvine, California, United States; e University of California, Irvine, Sue and Bill Gross Stem Cell Research Center, Irvine, California, United States; f University of Dayton, Department of Physics, Dayton, Ohio, United States; g Universidad de Málaga, Instituto de Investigacion Biomedica de Malaga y Plataforma en Nanomedicina-IBIMA Plataforma BIONAND, Facultad de Ciencias, Departamento Biologia Celular, Genetica y Fisiologia, Malaga, Spain; h Instituto de Salud Carlos III, CIBER de Enfermedades Neurodegenerativas (CIBERNED), Madrid, Spain; i University of California, Riverside, School of Medicine, Division of Biomedical Sciences, Riverside, California, United States; j University of California, Irvine, Department of Neurology, Irvine, California, United States; k University of California, Irvine, Department of Neurobiology & Behavior, Irvine, California, United States; l University of California, Irvine, Department of Anatomy & Neurobiology, Irvine, California, United States; m University of California, Irvine, Department of Neurological Surgery, Irvine, California, United States; n University of California, Irvine, Department of Surgery, Irvine, California, United States

**Keywords:** laser speckle contrast imaging, cerebral blood flow, Alzheimer’s disease, neurovascular coupling, microscopy, RNA sequencing

## Abstract

**Significance:**

Alzheimer’s disease (AD) is an age-related neurodegenerative disorder with cerebrovascular alterations contributing to cognitive decline. Assessing cerebrovascular changes in mouse models that mimic the human condition of late-onset, sporadic AD is important for better human applicability.

**Aim:**

To assess cerebrovascular changes in three mouse models: (1) 3xTg-AD; (2) the humanized amyloid-beta knock-in (hAβ-KI) mouse model of late-onset, sporadic AD; and (3) age-matched wild-type mice.

**Approach:**

We measured resting-state cerebral blood flow (CBF) and neurovascular coupling (NVC) using laser speckle imaging (LSI) and performed *ex vivo* analyses of gene expression and cerebrovascular structure using bulk ribonucleic acid sequencing and confocal microscopy, respectively.

**Results:**

Our study identifies specific cerebrovascular alterations in the hAβ-KI mouse model, including increased resting-state CBF, a shift toward smaller blood vessel diameters, impaired NVC, and transcriptomic changes related to metabolism and inflammation. Notably, we found that the increased resting-state CBF was primarily associated with female hAβ-KI mice.

**Conclusions:**

Our findings demonstrate that the hAβ-KI mouse model exhibits cerebrovascular alterations that warrant further investigation to uncover the underlying mechanisms. Expanding these studies could enhance our understanding of cerebrovascular alterations in AD and support the development of targeted therapeutic strategies.

## 1 Introduction

Alzheimer’s disease (AD) is an age-related neurodegenerative disorder characterized by alterations to numerous cellular processes that lead to cognitive impairment.^1^ Sex-specific differences are well documented in AD, with women comprising two-thirds of AD patients, having a greater lifetime risk of developing AD (1 in 5) compared with males (1 in 10),^2^ and experiencing faster cognitive decline.^3^ In addition, recent studies indicate that cerebrovascular changes contribute significantly to cognitive decline, independent of the two primary hallmarks of AD pathology, amyloid-beta (Aβ) plaques and neurofibrillary tangles (NFT).^4^^–^^6^ Notably, some models suggest that cerebrovascular dysfunction may precede the deposition of Aβ and NFT.^7^ Methods to study cerebrovascular alterations during aging and AD include resting-state cerebral blood flow (CBF), neurovascular coupling (NVC), and examining cerebrovascular structure. Evidence from AD patients and mouse models reports reduced resting-state CBF, coinciding with increased Aβ levels,^8^^–^^12^ along with decreased microvascular labeling.^12^ Furthermore, impaired NVC is associated with age-related cognitive impairment^13^ and precedes cognitive decline in AD.^14^ Collectively, these findings underscore the importance of investigating cerebrovascular contributions to AD and aging in both humans and preclinical models. To ensure translational relevance, it is crucial to use mouse models that closely replicate human pathology.

To advance treatment strategies and enhance our understanding of cerebrovascular changes in AD, mouse models that accurately reflect the disease’s progression in humans are essential. Unfortunately, most current mouse models focus on early-onset familial AD (FAD) through overexpression of genetic mutations, a rare form of the disease representing less than 1% of all AD cases.^15^ Although early-onset FAD is a devastating condition, there is a critical need to study sporadic, late-onset AD (LOAD), which accounts for 98% of AD cases.^4^^,^^5^ To investigate LOAD, new mouse models with knock-in (KI)/knock-out (KO) and CRISPR gene-editing technologies have been and are being developed to more faithfully reflect the human condition.^16^ One promising model is the humanized amyloid-beta knock-in (hAβ-KI) mouse, designed to serve as a platform model to investigate genetic, aging, and environmental factors driving AD pathology.^17^ Unlike most AD mouse models, which produce Aβ plaques early in life, the hAβ-KI model does not develop Aβ plaques but provides the precursors for amyloid pathology, more closely resembling the human condition. Furthermore, hAβ-KI mice exhibit age-dependent changes in behavior, synaptic plasticity, inflammatory response, and gene expression, mirroring the late-onset progression observed in sporadic human AD cases.^17^ However, the cerebrovascular changes in this model during the development of AD-like pathology remain unexplored.

This investigatory study was designed to explore cerebrovascular changes in the hAβ-KI mouse model (12 to 27 m.o.) compared with age-matched 3xTg-AD (a common mouse model with FAD mutations) and wild-type (WT) mice. We hypothesized that hAβ-KI mice would have impaired cerebrovascular structure and function through: decreased CBF with increasing age, reduced CBF in hAβ-KI compared with WT mice and a greater CBF reduction in 3xTg-AD mice, transcriptomic alterations that help explain CBF alterations, reduced vascular density and smaller vessel diameters in hAβ-KI mice, and impaired NVC in hAβ-KI mice. To test our hypotheses, we measured resting-state CBF and NVC using laser speckle imaging (LSI) and performed *ex vivo* analyses of gene expression and cerebrovascular structure using bulk RNA sequencing and confocal microscopy, respectively. Our results reveal alterations to the cerebrovasculature of hAβ-KI mice, including increasing CBF with age, higher resting-state CBF, a shift toward smaller blood vessel diameters (left-shifted distribution of vessel diameters), impaired NVC, and transcriptomic changes related to metabolism and inflammation compared with age-matched WT mice. Collectively, these findings demonstrate that the hAβ-KI mouse model exhibits cerebrovascular alterations that warrant further investigation to uncover the underlying mechanisms.

## 2 Materials and Methods

### 2.1 Experimental Cohorts and Animal Models

**Fig. 1:**
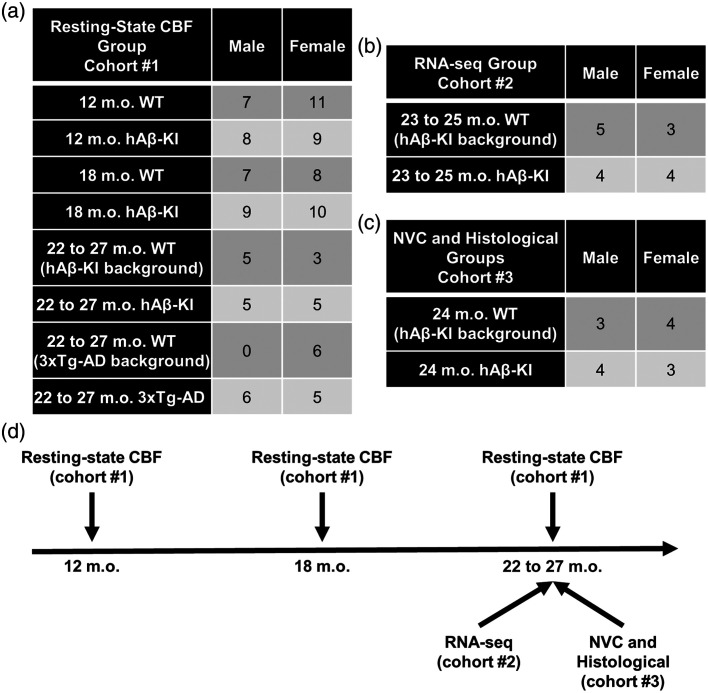
Experimental cohorts and timeline. (a) Mouse numbers used in cohort #1 to examine resting-state CBF in 12 and 18 m.o. WT and hAβ-KI mice from the same genetic background and 22 to 27 m.o. WT (hAβ-KI and 3xTg-AD mouse backgrounds), hAβ-KI, and 3xTg-AD mice. (b) Mouse numbers used in cohort #2 to examine RNA sequencing data in 23 to 25 m.o. WT (hAβ-KI background) and hAβ-KI mice. (c) Mouse numbers used in cohort #3 to examine NVC and cerebrovasculature in 24 m.o. WT (hAβ-KI background) and hAβ-KI mice. (d) Experimental timeline of the measurements performed for each cohort.

This study used three cohorts of mice. Figure 1 shows an outline of the cohorts and measurement time points used. Mice in cohort #1 had resting-state CBF measured across different age groups (12, 18, and 22 to 27 m.o.) to simulate midlife to late-life in humans and genotypes (WT, hAβ-KI, and 3xTg-AD). Mice in cohort #2 utilized WT and hAβ-KI mice at 23 to 25 m.o. and had bulk RNA-sequencing performed to examine transcriptomics. Mice in cohort #3 utilized WT and hAβ-KI mice at 24 m.o. to examine cerebrovascular structure and neurovascular coupling (NVC).

The MODEL-AD consortium at UC Irvine bred the mice used in this study, but mice are now commercially available. Two different sets of hAβ-KI mice were used. Cohorts #1 and #2 used hAβ-KI mice on a C57BL/6NJ congenic background (JAX Stock No. 032013). Second, cohort #3 used hAβ-KI mice on a mixed C57BL/6J and C57BL/6NJ background (JAX Stock No. 030898).^17^ In cohort #3, two hAβ-KI mice were unable to complete NVC and one hAβ-KI mouse was not able to complete vascular labeling. Cohort #1 also used homozygous 3xTg-AD mice on a B6; 129 genetic background.^18^^,^^19^ The IACUC at UC Irvine approved all relevant ethical regulations and procedures.

### 2.2 Animal Preparation

Each mouse undergoing optical imaging was anesthetized with 4% isoflurane balanced with 100% $O_{2}$. Mice were then placed in a stereotactic frame, and isoflurane was decreased to 2% balanced room air for surgery and resting-state CBF *in vivo* optical imaging. For NVC measurements, isoflurane was decreased to 1.2% for 10 min before starting data acquisition. Body temperature was maintained at 37°C with a feedback heating pad (Harvard Apparatus, Holliston, Massachusetts, United States). To allow for optical imaging, we used a previously described method to measure cortical CBF using an intact skull preparation.^20^ The scalp was retracted to enable visualization of brain regions between lambda and bregma. The overlying fascia was removed. The exposed skull was hydrated with saline, and a glass cover slip was placed on top to maintain skull hydration and transparency. This preparation enabled optical imaging of cortical CBF and NVC through an intact skull.

### 2.3 Resting-State CBF Measurement

We measured resting-state CBF using a previously described laser speckle imaging (LSI) system.^21^^–^^23^ Briefly, a stabilized 809 nm laser (Coherent, Saxonburg, Pennsylvania, United States) with a long coherence length was expanded to illuminate the cortical surface through the intact skull preparation. Raw speckle images of the backscattered light were collected with a 10-ms exposure time at 60 fps using a FLIR BlackflyS camera (Teledyne FLIR, Wilsonville, Oregon, Unites States). Cross-polarization was used to minimize specular reflection.

We obtained resting-state CBF from 60 raw speckle images using the temporal speckle contrast algorithm.^24^^,^^25^ The temporal algorithm was selected to minimize effects due to static scattering from the skull.^24^^,^^26^ We used a previously described protocol that removed motion artifact images to calculate the temporal speckle contrast.^25^^,^^27^ Temporal speckle contrast images were converted to CBF maps using a simplified speckle imaging equation $\mathrm{CBF}=1/(2TK^{2})$, where T is the exposure time, and K is the speckle contrast.^28^^,^^29^ β, a normalization factor to account for speckle averaging due to a mismatch of speckle size and detector size, polarization, and coherence effects,^30^ was measured from a static phantom to minimize day-to-day variability, resulting in a beta-corrected $\mathrm{CBF}=\beta /(2TK^{2})$.^31^^–^^37^

We used the beta-corrected CBF maps to quantify resting-state CBF. The median resting-state CBF was calculated from a manually drawn global ROI over the right hemisphere and used for statistical comparisons. Comparisons were performed using one-way ANOVA or two-way ANOVA with *post hoc* Tukey or Sidak multiple comparisons test. The specific test used for each figure is listed in the figure captions. A p<0.05 was considered statistically significant, and a p<0.15 was considered trending.

### 2.4 Gene Expression Through Bulk RNA Sequencing

To prepare tissue for gene expression analysis, mice were perfused with 10 mL of sterile saline. Brains were microdissected, snap-frozen, and stored in a −80°C freezer until RNA isolation. Bulk RNA sequencing of the cortex was performed. An RNeasy Micro kit (Qiagen, Hilden, Germany) was used per manufacturer’s guidelines to isolate RNA from cells in the cortex. Sequencing was performed by the Genomics Research and Technology Hub (GRT Hub) at UC Irvine. RNA integrity was measured using the Bioanalyzer Agilent 2100. Five hundred nanograms of RNA per sample was used to create RNA-seq libraries through the Illumina TruSeq mRNA stranded protocol. Each sample was then sequenced on the Illumina HiSeq 4000 platform with ∼30  M reads per sample.

RNA sequencing read integrity was verified using FastQC. BBDuk was used to trim adapters and filter out poor-quality reads.^38^ Reads were pseudoaligned to the Ensembl GRCm39 mouse transcriptome using Kallisto.^39^ Differential gene expression analysis was conducted in R using the DESeq2 package.^40^ Lowly expressed genes with expression counts summed over all samples <10 were removed before differential expression analysis. Differential gene expression was analyzed between WT and hAβ-KI (males and females pooled). Differentially expressed genes were identified with a false discovery rate (FDR)<0.1 and p<0.05. Data visualization methods included heat maps and volcano plots to highlight significantly upregulated and downregulated genes.

### 2.5 Vascular Labeling and *Ex Vivo* Microscopy

To assess the cerebral microvasculature, confocal microscopy was used to visualize blood vessels labeled with lectin-DyLight-649. Mice in cohort #3 were injected retro-orbitally with a 200  μL solution of lectin-DyLight-649 diluted in sterile saline (0.25  mg/mL) (Vector Laboratories, Burlingame, California, United States). The solution was allowed to circulate for 20 min. Cardiac perfusion with serial administration of saline and 10% formalin was performed for 5 min each at 2  mL/min. Hemibrains were stored within 10% formalin overnight and subsequently within 0.01% sodium azide in PBS until sectioning.

One 1-mm-thick coronal section from the hemibrain contralateral to the hindpaw stimulation side for NVC measurements was used for analysis. Each section was cleared using a modified iDISCO (immunolabeling-enabled three-dimensional imaging of solvent-cleared organs) protocol.^41^ Briefly, samples were dehydrated in a series of 20-min methanol washes (20%, 40%, 60%, 80%, 100%, and 100%) and immersed in a solution of 66% dichloromethane and 33% methanol for 1 h. Finally, sections were immersed twice in dichloromethane for 15 min each and stored in dibenzyl ether for refractive index matching.

Brain sections were imaged using a confocal microscope (True Confocal Scanner SP8, Leica Biosystems, Illinois, United States) with a 10x/0.3 NA HC PL Fluotar objective. The microscope used a 633-nm HeNe laser to excite the lectin-DyLight-649 bound to the brain vasculature, with emission captured between 638 and 783 nm. Confocal image stacks were reduced to maximum intensity projections (MIPs) of ∼70-μm-thick tissue sections for region of interest (ROI) selection. The number of MIPs varied between 7 and 12 for all mice. Three readers were each tasked with manually tracing an ROI around the cortex. This was performed for multiple MIPs that spanned each brain section. A consensus ROI was determined using a majority filter applied to the ROIs identified by each reader.

Custom-written MATLAB (MathWorks, Natick, Massachusetts, United States) code was written to process cerebrovascular images. Within each ROI, images were binarized using an automated iterative selection method to identify the intensity threshold.^42^^,^^43^ A size threshold was used to remove continuous vessel segments smaller than $51\;{\mu m}^{2}$. Each ∼70-μm-thick MIP was skeletonized. Due to the variable number of MIPs for each mouse, the five MIPs within each mouse that had the longest vessel lengths were used to calculate vessel density. Vessel density was calculated as the total vessel length divided by the total cortical area across the five MIPs selected for vessel density calculation. A t-test was performed with a p<0.05 statistically significant. To test if the distributions of vessel diameters were different between WT and hAβ-KI mice, a histogram of vessel diameters was obtained. The histogram counts for each vessel diameter were normalized to the vessel diameter with the most counts. To ensure longer vessels did not skew the results, the mean vessel diameter along each vessel segment was used. The normalized vessel diameter distributions between WT and hAβ-KI mice were compared using a Kolmogorov–Smirnov test, with significant differences between distributions identified with a p<0.05. One mouse (1 M, hAβ-KI) was excluded as the labeling procedure could not be completed.

### 2.6 Hindpaw Stimulation to Assess Neurovascular Coupling

We assessed NVC using LSI. A stabilized 633 nm laser (Coherent, Saxonburg, Pennsylvania, United States) was expanded to illuminate the cortical surface through the intact skull preparation.^44^ Raw speckle images of the backscattered light were collected with a 10-ms exposure time at 50 fps using a FLIR BlackflyS camera (Teledyne FLIR, Wilsonville, Oregon, United States). Cross-polarization was used to minimize specular reflection. We assessed NVC in response to hindpaw stimulation.^45^^–^^47^ Two electrodes were placed in the right hindpaw between the first and second digits and the fourth and fifth digits. Forty 15 s trials were performed. Trials included a 2-s baseline period, 2-s stimulation period, and 11-s recovery period. The stimulation sequence used an isolated stimulator (World Precision Instruments, Sarasota, Florida, United States) delivering 500  μs pulses at 4 Hz with ∼1  mA. A data acquisition board and LabVIEW control software (NI, Austin, Texas, United States) triggered the stimulation sequence using TTL pulses and time-synced LSI data with the stimulation sequence.

Each raw speckle image was converted to a CBF map. CBF maps were downsampled into 15×15 windows to decrease memory, reduce calculation time, and improve signal-to-noise. Within each window, the average CBF value was calculated to improve signal to noise. For each trial, time courses from the top 10% of downsampled pixels with the largest response during stimulation were averaged together. The percent change in CBF was calculated for each trial using the 2-s baseline period for normalization. Then, all trials were averaged together to obtain a single, final percent change in the CBF time course. The final percent change in the CBF time course was used to quantify the NVC response using the maximum percent change in CBF and the area under the curve of the CBF during hindpaw stimulation. A t-test was performed for each comparison with a p<0.05 statistically significant. Two mice (2 M, hAβ-KI) were excluded because data acquisition could not be completed.

## 3 Results

### 3.1 LSI-Measured Resting-State CBF Increases with Age in hA***β***-KI Mice

**Fig. 2:**
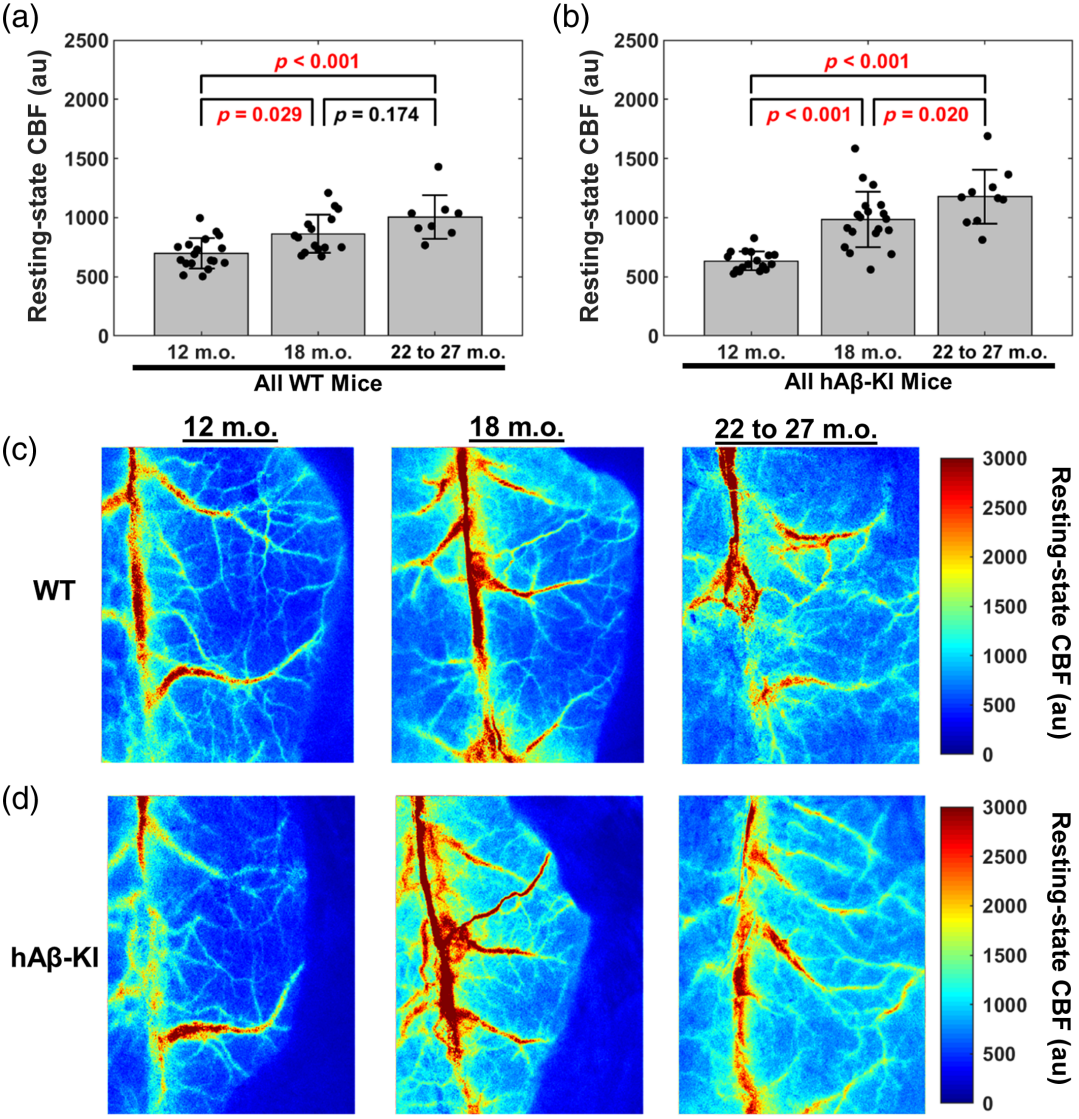
hAβ-KI mice have increased resting-state CBF, whereas WT mice trend toward increased resting-state CBF with increasing age. Comparing resting-state CBF between 12, 18, and 22 to 27 m.o. (a) WT and (b) hAβ-KI mice (two-way ANOVA, Tukey *post hoc* test). Representative images from (c) wild type and (d) hAβ-KI mice, where the mice selected had the median resting-state CBF from each age and genotype. P-values are shown for each comparison, with a p<0.05 considered statistically significant (red text).

We employed LSI to assess cortical, resting-state CBF at 12, 18, and 22 to 27 m.o. hAβ-KI and age-matched WT control mice (males and females pooled) (Fig. 2). In WT mice, resting-state CBF increased from 12 to 18 m.o. (p=0.029) and 12 to 22–27 m.o. (p<0.001); however, resting-state CBF had a non-significant difference from 18 to 22–27 m.o. (p=0.174) [Fig. 2(a)]. In hAβ-KI mice, resting-state CBF increased from 12 to 18 m.o. (p<0.001), 12 to 22–27 m.o. (p<0.001), and 18 to 22–27 m.o (p=0.020) [Fig. 2(b)]. Figures 2(c) and 2(d) show representative CBF maps from the median mouse of WT and hAβ-KI mice in the 12, 18, and 22 to 27 m.o. groups. Collectively, these data suggest that resting-state, cortical CBF increases with aging in hAβ-KI mice and trends toward increased cortical CBF in WT mice.

### 3.2 Trends Toward Sex-Specific Differences in hA***β***-KI Mice Using LSI-measured Resting-State CBF

**Fig. 3:**
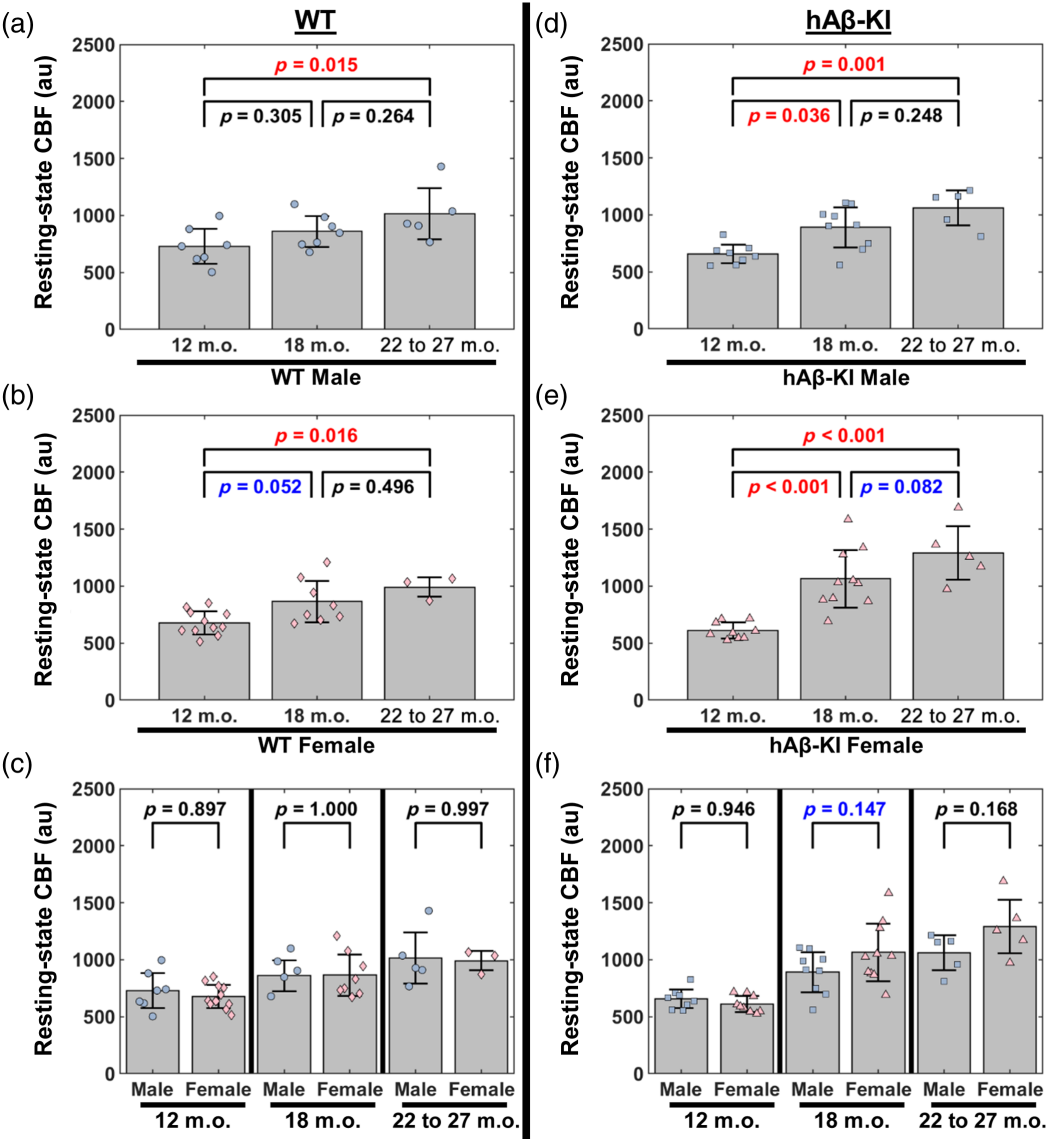
Male and female hAβ-KI mice have differing resting-state CBF trajectories compared with male and female WT mice. (a)–(c) (left) Comparing resting-state CBF between 12, 18, and 22 to 27 m.o. for (a) male, WT and (b) female, WT mice (two-way ANOVA, Tukey *post hoc* test). (c) Comparing resting-state CBF at 12, 18, and 22 to 27 m.o. between male and female WT mice (two-way ANOVA, Sidak *post hoc* test). (d)–(f) (right) Comparing resting-state CBF between 12, 18, and 22 to 27 m.o. for (d) male, hAβ-KI and (e) female, hAβ-KI mice (two-way ANOVA, Tukey *post hoc* test). (f) Comparing resting-state CBF at 12, 18, and 22 to 27 m.o. between male and female hAβ-KI mice (two-way ANOVA, Sidak *post hoc* test). P-values are shown for each comparison, with a p<0.05 considered statistically significant (red text) and p<0.15 considered trending (blue text).

We next assessed age-based comparisons within each sex for hAβ-KI and age-matched WT control mice (Fig. 3). Male WT mice had increased resting-state CBF from 12 to 22–27 m.o. (p=0.015); however, resting-state CBF had non-significant differences from 12 to 18 m.o. (p=0.305) and 18 to 22–27 m.o. (p=0.264) [Fig. 3(a)]. Female WT mice had increased resting-state CBF from 12 to 22–27 m.o. (p=0.016) and an increasing trend from 12 to 18 m.o. (p=0.052); however, resting-state CBF had a non-significant difference from 18 to 22–27 m.o. (p=0.496) [Fig. 3(b)]. Comparing male and female WT mice at 12, 18, and 22–27 m.o., there were no significant differences or trends for resting-state CBF [Fig. 3(c)].

Male hAβ-KI mice had increased resting-state CBF from 12 to 18 m.o. (p=0.036) and 12 to 22–27 m.o. (p=0.001); however, resting-state CBF had a non-significant difference from 18 to 22–27 m.o. (p=0.248) [Fig. 3(d)]. Female hAβ-KI mice had increased resting-state CBF from 12 to 18 m.o. (p<0.001) and 12 to 22–27 m.o. (p<0.001), and an increasing trend from 18 to 22–27 m.o. (p=0.082) [Fig. 3(e)]. Comparing male and female hAβ-KI mice at 12, 18, and 22 to 27 m.o., there were no significant differences; however, female hAβ-KI trended toward elevated resting-state CBF compared with male hAβ-KI mice at 18 m.o. (p=0.147) [Fig. 3(f)].

### 3.3 hA***β***-KI Mice Have Higher Resting-State CBF Compared with WT and 3xTg-AD Mice

**Fig. 4:**
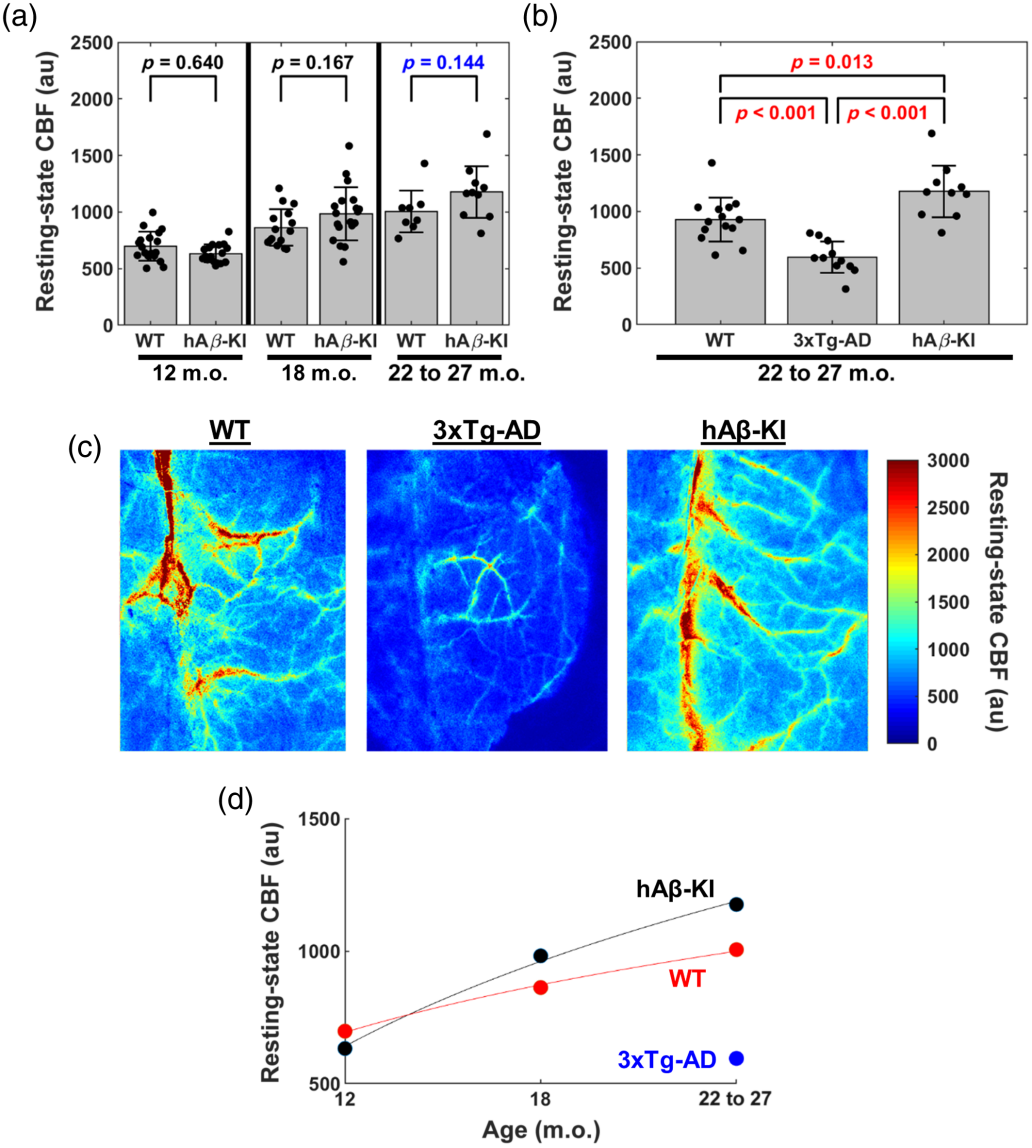
Assessing resting-state CBF differences among hAβ-KI, WT, and 3xTg-AD mice. (a) Comparing resting-state CBF between WT and hAβ-KI mice at 12, 18, and 22 to 27 m.o. (two-way ANOVA, Sidak *post hoc* test). (b) Comparing resting-state CBF between WT, 3xTg-AD, and hAβ-KI mice at 22 to 27 m.o. (one-way ANOVA, Tukey *post hoc* test). (c) Representative images from (left) wild type, (middle) 3xTg-AD, and (right) hAβ-KI mice, where the mice selected had the median resting-state CBF from each genotype. (d) Summarizing the temporal evolution of CBF in WT, hAβ-KI, and 3xTg mice by fitting a logarithmic curve through the mean CBF of each group. Solid lines are to only guide the eye because we do not have data at all these ages. P-values are shown for each comparison, with a p<0.05 considered statistically significant (red text) and p<0.15 considered trending (blue text).

We next compared resting-state CBF between hAβ-KI and age-matched WT control mice (males and females pooled) at 12, 18, and 22 to 27 m.o. hAβ-KI trended toward higher resting-state CBF than age-matched WT control mice at 22 to 27 m.o. (p=0.144); however, there were no significant differences or trends in resting-state CBF between WT and hAβ-KI at 12 m.o. (p=0.640) and 18 m.o. (p=0.167) [Fig. 4(a)]. To place these observations in context, we next studied resting-state CBF in 3xTg-AD mice at 22 to 27 m.o. and compared the data with our hAβ-KI and WT measurements. hAβ-KI mice had increased resting-state CBF compared with 3xTg-AD (p<0.001) and WT (p=0.013) mice at 22 to 27 m.o., whereas 3xTg-AD mice had decreased resting-state CBF compared with WT mice (p<0.001) [Fig. 4(b)]. WT mice with hAβ-KI and 3xTg-AD backgrounds were pooled as there was a non-significant trend in resting-state CBF (p=0.090). Figure 4(c) shows representative CBF maps from the median mouse of each group. Figure 4(d) summarizes the temporal evolution of CBF in WT, hAβ-KI, and 3xTg mice by fitting a logarithmic curve through the mean CBF of each group.

### 3.4 Resting-State CBF Trends in hA***β***-KI Mice are Driven by Female Mice, Not Male Mice

**Fig. 5:**
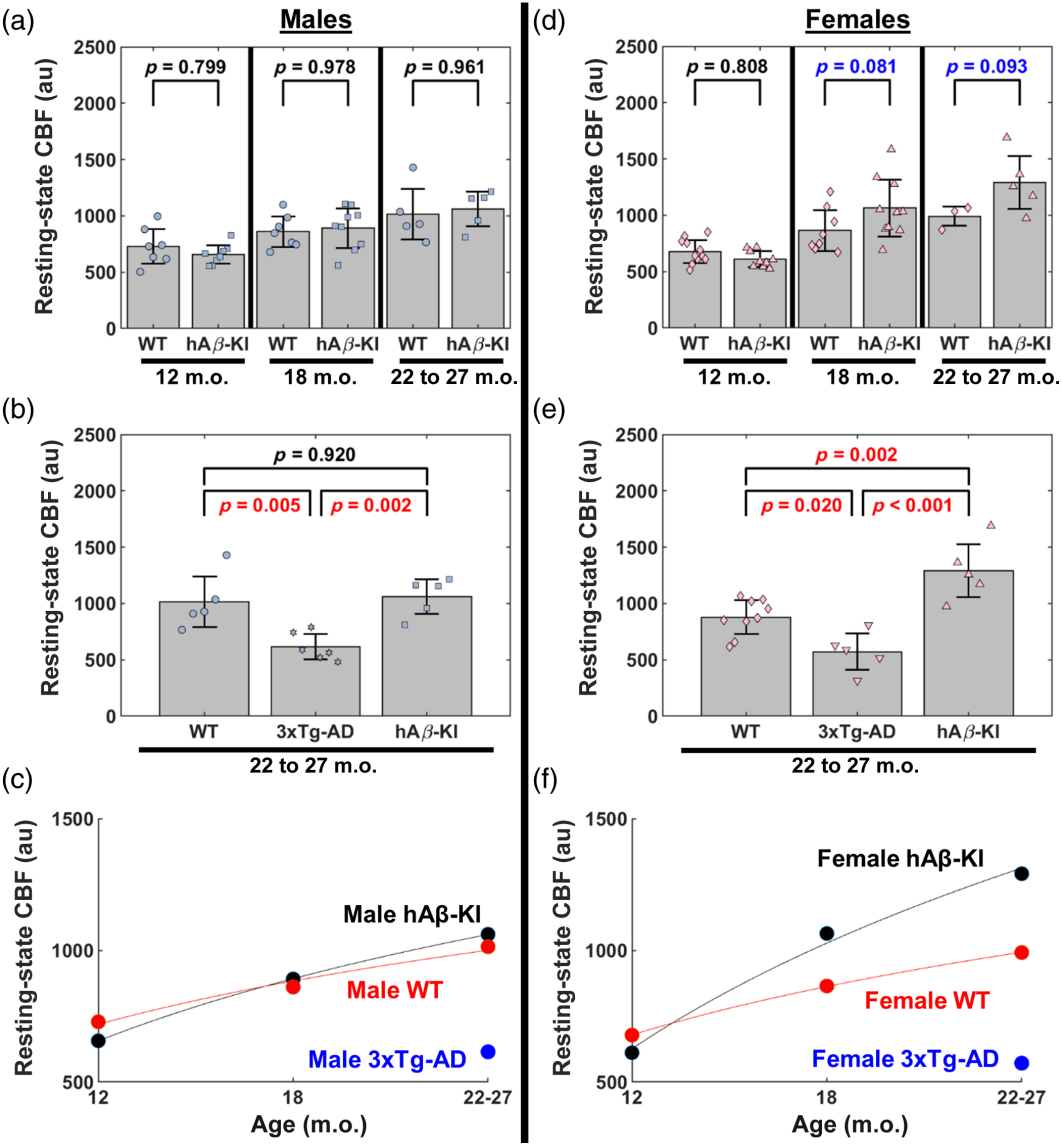
Male and female 3xTg-AD mice have reduced resting-state CBF, whereas female hAβ-KI mice drive resting-state CBF trends compared to WT mice. (a)–(c) (left) Resting-state CBF in male mice. (a) Comparing resting-state CBF between WT and hAβ-KI male mice at 12, 18, and 22 to 27 m.o. (two-way ANOVA, Sidak *post hoc* test) (b). Comparing resting-state CBF between WT, 3xTg-AD, and hAβ-KI male mice at 22 to 27 m.o. (two-way ANOVA, Tukey *post hoc* test). (c) Summarizing the temporal evolution of CBF in WT, hAβ-KI, and 3xTg male mice by fitting a logarithmic curve through the mean CBF of each group. Solid lines are to only guide the eye because we do not have data at all these ages. (d)–(f) (right) Resting-state CBF in female mice. (d) Comparing resting-state CBF between WT and hAβ-KI female mice at 12, 18, and 22 to 27 m.o. (two-way ANOVA, Sidak *post hoc* test). (e) Comparing resting-state CBF between WT, 3xTg-AD, and hAβ-KI female mice at 22 to 27 m.o. (two-way ANOVA, Tukey *post hoc* test). (f) Summarizing the temporal evolution of CBF in WT, hAβ-KI, and 3xTg female mice by fitting a logarithmic curve through the mean CBF of each group. Solid lines are to only guide the eye because we do not have data at all these ages. P-values are shown for each comparison, with a p<0.05 considered statistically significant (red text) and p<0.15 considered trending (blue text).

We next assessed age-based comparisons within each sex between hAβ-KI and age-matched WT. There were no significant differences or trends in resting-state CBF between male WT and male hAβ-KI mice at 12 m.o. (p=0.799), 18 m.o. (p=0.978), and 22 to 27 m.o. (p=0.961) [Fig. 5(a)]. There was a non-significant difference in resting-state CBF between female WT and female hAβ-KI mice at 12 m.o. (p=0.808); however, there was a trend towards increased resting-state CBF in females at 18 m.o. (p=0.081) and 22 to 27 m.o. (p=0.093) [Fig. 5(d)].

We next focused on the 22 to 27 m.o. cohort to compare resting-state CBF within each sex between 3xTg-AD, hAβ-KI, and age-matched WT (we pooled WT mice from 3xTg-AD and hAβ-KI genetic backgrounds as there was no significant difference in resting-state CBF, p=0.090) mice [Figs. 5(b) and 5(e)]. Male 3xTg-AD mice had significantly lower resting-state CBF compared to male hAβ-KI (p=0.002) and male WT (p=0.005) mice, whereas male hAβ-KI mice did not have significantly different resting-state CBF compared to male WT mice (p=0.920) [Fig. 5(b)]. Similar to male 3xTg-AD mice, resting-state CBF in female 3xTg-AD mice was significantly lower compared with female hAβ-KI (p<0.001) and female WT (p=0.020) mice. Interestingly, resting-state CBF in female hAβ-KI was significantly higher compared with female WT mice (p=0.002) [Fig. 5(e)]. Furthermore, male and female 3xTg-AD mice had no difference in resting-state CBF (p=0.976). Figures 5(c) and 5(f) summarize the temporal evolution of CBF in WT, hAβ-KI, and 3xTg mice for each sex by fitting a logarithmic curve through the mean CBF of each group.

### 3.5 Transcriptomic Alterations in the Cortex of hA***β***-KI Mice

**Fig. 6:**
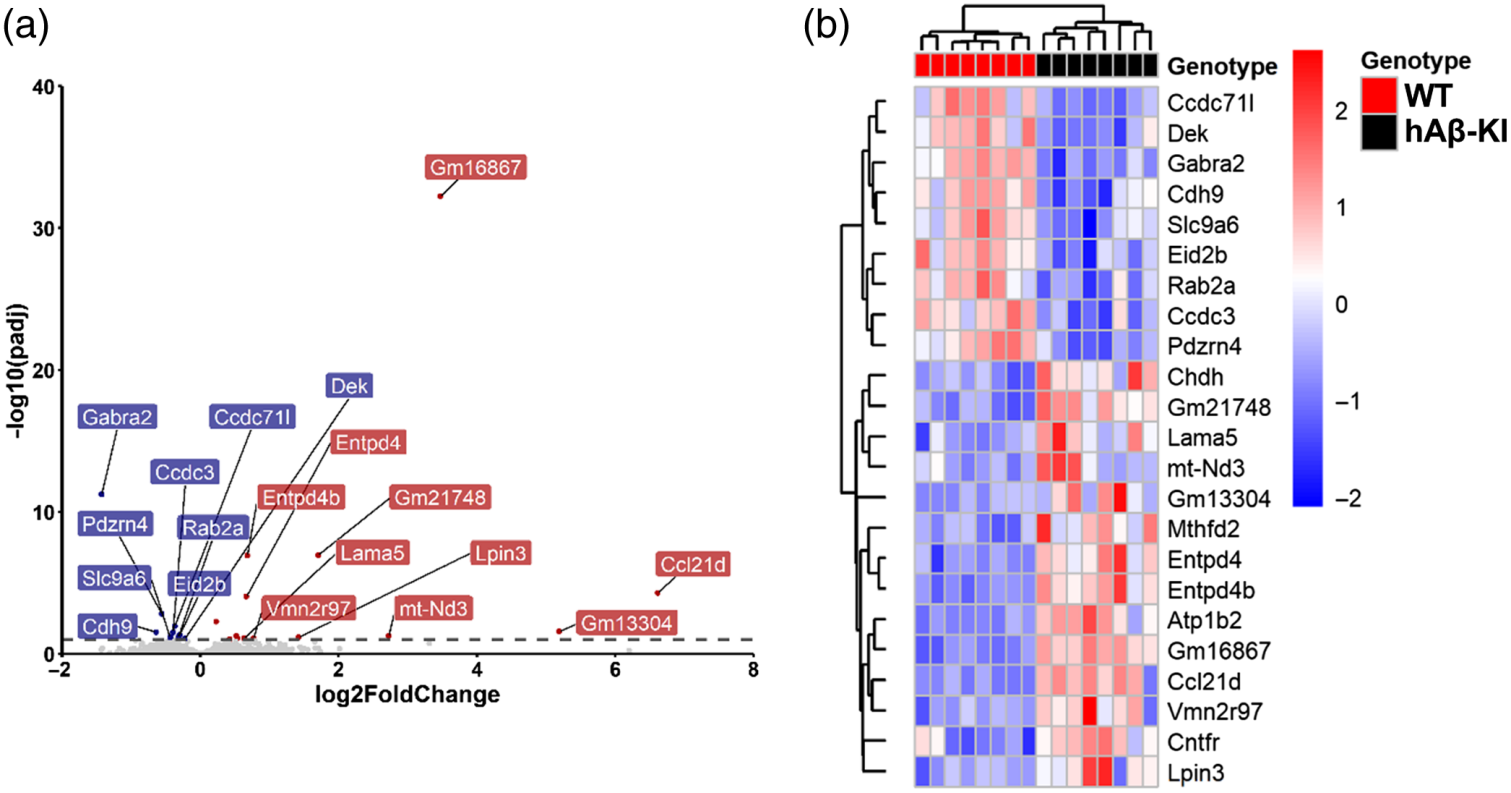
Transcriptomic differences between 23 and 25 m.o. WT and hAβ-KI mice. (a) Volcano plot and (b) heatmap showing differentially expressed genes with p<0.05 and FDR<0.1. Blue shows downregulated gene expression, and red shows upregulated gene expression.

To investigate whether gene expression changes could explain the elevated resting-state CBF observed in hAβ-KI mice, we conducted bulk RNA-seq on transcripts extracted from cortical tissue (Fig. 6). This analysis identified 23 genes that were differentially expressed in hAβ-KI mice compared to age-matched WT controls, specifically between 23 and 25 m.o. (cohort #2). The differentially expressed genes are involved in metabolism (*mt-Nd3*, *Chdh*, *Entpd4b*, *Entpd4*, *Atp1b2*), neuroplasticity and neurotransmission (*Atp1b2*, *Gabra2*), and inflammation (*Ccdc3*, *Ccdc71l*, *Eid2b*, *Ccl21d*). These changes in gene expression may indirectly contribute to the observed elevation in resting-state CBF by promoting increased metabolic activity and heightened inflammatory responses.

### 3.6 hA***β***-KI and WT Mice Have Different Vessel Diameter Distributions

**Fig. 7:**
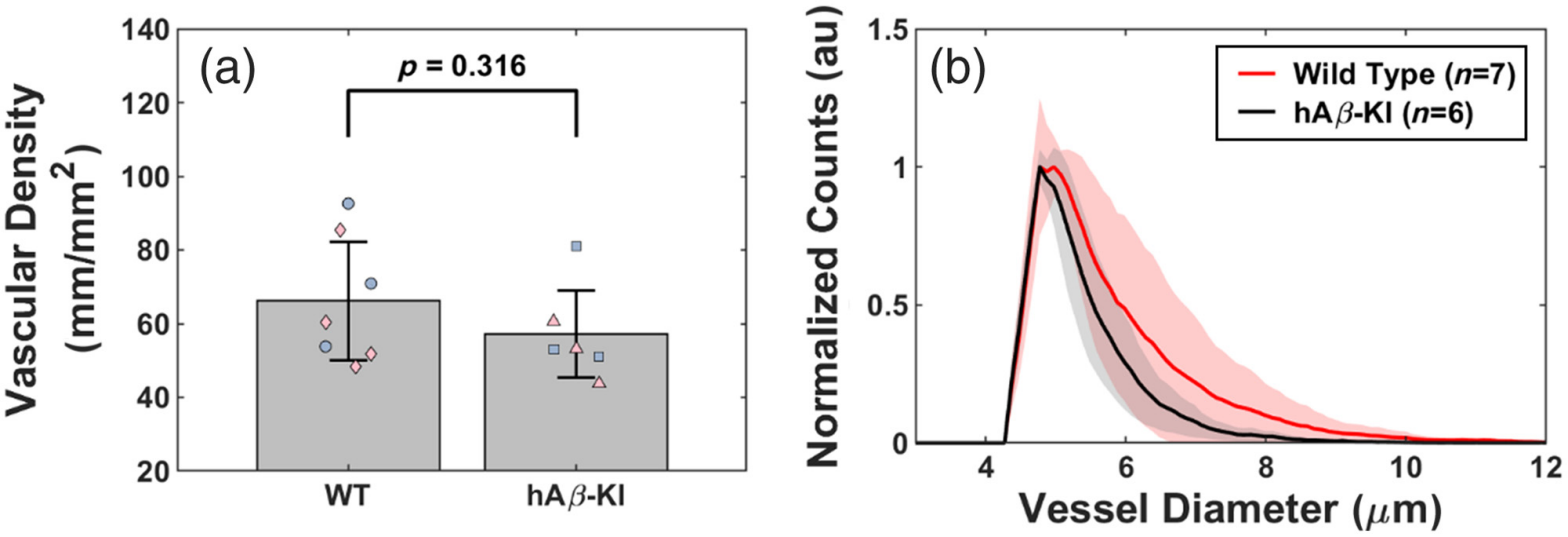
Comparison of cerebrovascular structure between 24 m.o. WT and hAβ-KI mice. (a) Comparing the vascular density between WT (blue circle = male; pink diamond = female) and hAβ-KI (blue square = male; pink triangle = female) mice (t-test). (b) Distribution of vessel diameters in WT (red, n=7) and hAβ-KI (black, n=6) mice. Shaded areas represent the standard deviation across all mice within each respective group.

To assess changes in cerebrovascular structure that may possibly describe the alterations we observe in resting-state CBF, we used confocal microscopy of optically cleared brain sections in 24 m.o. hAβ-KI and WT mice (cohort #3). There were no differences in the vascular density [Fig. 7(a), p=0.316]. However, the distribution of vessel diameters (<12  μm) in hAβ-KI mice was left-shifted compared with WT mice [Fig. 7(b), p<0.001]. Together with the resting-state CBF data, the left-shifted vessel diameter data would suggest that the red blood cell speed through blood vessels may be higher to account for the trend towards increased resting-state CBF in hAβ-KI mice.

### 3.7 hA***β***-KI Mice Have Impaired Neurovascular Coupling

**Fig. 8:**
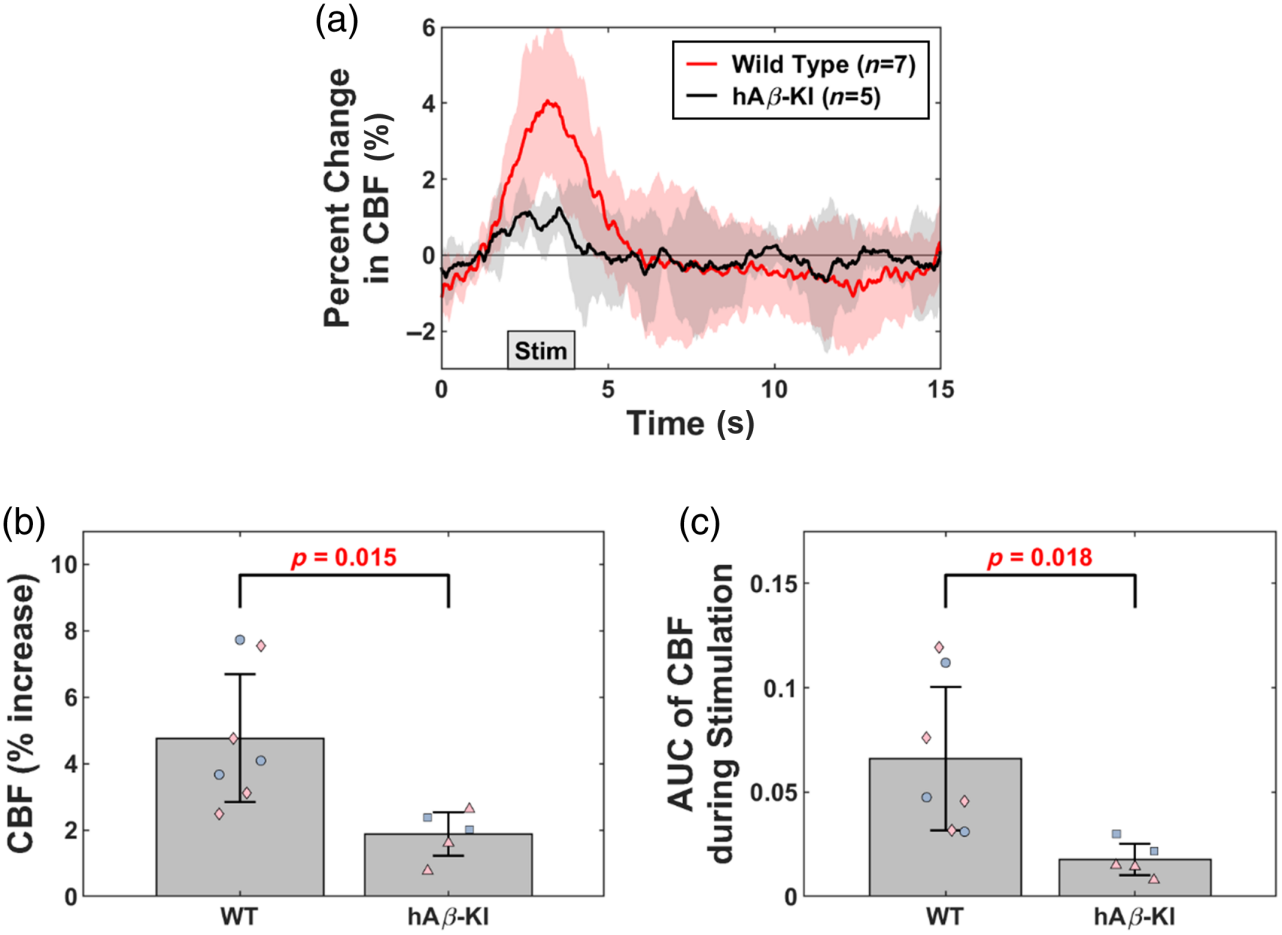
hAβ-KI mice have altered neurovascular coupling (NVC). (a) Averaged percent change in CBF during hindpaw stimulation to assess NVC in WT (red, n=7) and hAβ-KI (black, n=5) mice. Shaded areas represent the standard deviation across all mice within each respective group. (b) Comparing the maximum percent increase in CBF due to hindpaw stimulation between WT (blue circle = male; pink diamond = female) and hAβ-KI (blue square = male; pink triangle = female) mice (t-test). (c) Comparing the area under the curve (AUC) of CBF during hindpaw stimulation between WT (blue circle = male; pink diamond = female) and hAβ-KI (blue square = male; pink triangle = female) mice (t-test). P-values are shown for each comparison, with a p<0.05 considered statistically significant (red text).

To assess changes in neurovascular function, we assessed neurovascular coupling (NVC) in 24 m.o. hAβ-KI and WT mice (cohort #3). Compared with WT mice, hAβ-KI mice had a reduced NVC response due to hindpaw stimulation [Fig. 8(a)]. hAβ-KI mice had a lower peak percent increase during stimulation [p=0.015, Fig. 8(b)] and less total perfusion during stimulation than age-matched WT mice [p=0.018, Fig. 8(c)]. In addition, to minimize any potential influence of differences in resting-state CBF on NVC data, we examined the overall CBF change (ΔCBF) without normalization (Fig. S1 in the Supplementary Material). Percent change in CBF and ΔCBF results agree, demonstrating impaired NVC in hAβ-KI mice. However, temporal dynamics did not differ between WT and hAβ-KI mice (Fig. S2 in the Supplementary Material).

## 4 Discussion

This study demonstrates cerebrovascular alterations in the hAβ-KI mouse model, which exhibits increased resting-state CBF, a left-shifted distribution of vessel diameters, and impaired NVC. Transcriptomic alterations in hAβ-KI mice suggest potential mechanisms that may contribute to the elevated resting-state CBF observed. Notably, we found that these changes in resting-state CBF are driven predominantly in female mice rather than in male mice, indicating possible sex-specific differences. Although the resting-state CBF and cerebrovascular structure showed mild alterations, the NVC response in hAβ-KI mice was profoundly reduced. These findings highlight cerebrovascular changes in the hAβ-KI model, underscoring the need for further investigating the underlying mechanisms and their relevance to AD pathology.

### 4.1 Resting-State CBF Across Aging and Sex Differences

Our resting-state CBF data show that hAβ-KI mice exhibit an age-dependent increase in cortical resting-state CBF from 12 to 18 m.o. and 18 to 22–27 m.o. [Fig. 2(b)]. In comparison, age-matched WT mice showed an increase from 12 to 18 m.o. [Fig. 2(a)]. In human studies, however, resting-state CBF generally decreases with age,^48^^–^^50^ with an accelerated reduction in AD patients.^51^ Although studies in mouse models across midlife (12 m.o.) to older age (24 m.o.) are limited, some mouse studies suggest conflicting trends. For example, Moeini et al.^52^ observed an inverted U-shaped pattern in C57BL6/J mice, with CBF increasing from young (6 to 8 m.o.) to middle (13 to 15 m.o.) age and then decreasing in older (24 to 26 m.o.) mice, particularly in arterioles compared to venules. Conversely, a longitudinal study in female C57BL6/J mice demonstrated a continuous increase in resting-state CBF throughout aging,^53^ consistent with our findings.

Our study also revealed sex-specific differences, with female hAβ-KI mice exhibiting a trend towards increased resting-state CBF at 18 m.o. compared with males [Fig. 3(f)]. A previous study also showed 18 m.o. female hAβ-KI mice, but not male mice, had distinct microbiome changes compared to WT mice,^54^ which underscores the importance of investigating sex-specific differences. The observation that females tend to have higher resting-state CBF than males aligns with previously reported human data.^49^^,^^50^^,^^55^^,^^56^ Although the precise mechanisms driving this sex difference in hAβ-KI mice remain unclear, our findings suggest that sex may play a significant role in cerebrovascular dynamics and warrant further investigation to explore its underlying causes.

### 4.2 Unexpected Elevated Resting-State CBF and Clinical Relevance

Interestingly, our data showed that hAβ-KI mice, particularly females, challenge the general consensus that AD is associated with reduced CBF.^57^^–^^60^ Our original hypothesis was based on prior studies demonstrating decreased CBF in AD mouse models and patients, attributed to factors such as stalled neutrophils in capillaries,^61^ Aβ-induced capillary constriction through signaling to pericytes,^8^ and reduced vascular density.^62^ Our positive-control data in 22 to 27 m.o. 3xTg-AD mice (Fig. 4) confirm an expected decrease in resting-state CBF^12^ that we did not observe in hAβ-KI mice. Interestingly, hAβ-KI mice with the APOE4 allele had increased CBF at 18 m.o. compared with WT mice,^63^ similar to the hAβ-KI data presented in this study. Furthermore, some studies show instances where resting-state CBF elevation occurs in the preclinical phases of AD, particularly in cognitively normal human subjects with Aβ accumulation.^64^^,^^65^ These findings suggest that elevated resting-state CBF could represent an early compensatory mechanism before cognitive impairment develops. This interpretation is supported by data showing that hAβ-KI mice exhibit cognitive changes starting around 14 m.o.,^17^ placing them in a preclinical or MCI phase of AD. Therefore, the observed resting-state CBF elevation in hAβ-KI mice might reflect early, compensatory cerebrovascular changes that could have clinical implications for understanding early-stage AD development. Importantly, additional studies in older hAβ-KI mice may be critical to ensure that an expected decrease in resting-state CBF occurs, as is observed in humans.

### 4.3 Gene Expression Analysis Suggests Metabolic and Inflammatory Contributions

To further explore the potential mechanisms behind the increased resting-state CBF, we conducted bulk RNA sequencing to examine gene expression changes in hAβ-KI mice. Our data revealed upregulation in metabolic genes, including *mt-Nd3*, *Chdh*, *Entpd4b*, *Entpd4*, and *Atp1b2* (Fig. 6), which could drive increased resting-state CBF to meet higher metabolic demands.^66^^–^^69^ In human and animal studies, increased metabolic activity has been associated with elevated CBF as the brain compensates to fulfill energy requirements. In addition, we observed altered inflammatory-related genes (*Ccdc3*, *Ccdc71l*, *Eid2b*, and *Ccl21d*) that promote inflammation. Higher pro-inflammatory markers were previously observed in hAβ-KI mice,^17^^,^^70^ consistent with our data. Although inflammation typically reduces resting-state CBF,^71^ one inflammatory gene, *Ccl21d*, is associated with angiogenesis, which might offset some resting-state CBF reductions and contribute to the overall resting-state CBF increase observed in hAβ-KI mice. Thus, the interplay between metabolic and inflammatory genes may contribute to the cerebrovascular phenotype observed in hAβ-KI mice. Further analysis to assess Gene Ontology through gene set enrichment analysis (GSEA)^72^^,^^73^ revealed downregulated pathways related to neurotransmission through Gene Ontology molecular function, cellular component, and biological processes (Table S1 in the Supplementary Material). This downregulation does not appear to be related to the observed resting-state CBF changes, but may play a role in the impaired NVC observed in hAβ-KI mice (Fig. 8).

### 4.4 Cerebrovascular Structural Alterations and NVC Impairment

Histological analysis revealed a left-shifted distribution of vessel diameters in hAβ-KI mice compared with WT controls [Fig. 7(b)], although vascular density remained unchanged [Fig. 7(a)]. Assuming the same input pressure for all mice, the shift toward smaller vessel diameters may increase the speed of red blood cell flow based on fluid dynamics, potentially contributing to the elevated resting-state CBF measured by LSI. Previous studies indicate that LSI measurements can be sensitive to vessel diameter changes^74^ and may underestimate blood flow changes due to unaccounted tissue properties^75^ and LSI model limitations.^36^ Further studies are needed to confirm whether LSI underestimates CBF changes in hAβ-KI mice and to understand the influence of vessel structure on blood flow dynamics. In addition, hAβ-KI mice have changes in molecular $A\beta_{40}$ and $A\beta_{42}$ levels and OC+ granules associated with astrocytic processes.^17^ Because Aβ causes vasoconstriction via signaling to pericytes,^8^^,^^76^ this may explain the left-shifted vessel diameter distribution in hAβ-KI mice. Furthermore, astrocytes play a key role in regulating CBF,^77^^,^^78^ and Aβ-induced alterations in astrocyte function may impact resting-state CBF and NVC.

Our findings indicate a significant impairment in the NVC response in hAβ-KI mice (Fig. 8), a result that aligns with other AD mouse models displaying impaired NVC (ex: Tg2576,^79^^–^^81^ 3xTg-AD,^31^^,^^82^ and PS19^83^). Furthermore, human studies show impaired NVC during MCI and AD.^84^^,^^85^ These studies suggest that vascular alterations, such as cerebral amyloid angiopathy (CAA) or reduced nitric oxide production, are required for impaired NVC.^79^^–^^81^^,^^83^ However, the hAβ-KI model does not develop CAA. This suggests that the impaired NVC in hAβ-KI mice may be due to dysfunction within the neurovascular unit, particularly involving astrocytes and pericytes. Prior research indicates that Aβ oligomers can impair NVC^81^ through signaling pathways involving pericytes.^86^^,^^87^ In addition, astrocyte-associated OC+ granules may impair astrocyte ${\mathrm{Ca}}^{2+}$ signaling required for intact NVC.^84^^,^^88^ Furthermore, GSEA revealed downregulated pathways related to neurotransmission (Table S1 in the Supplementary Material), which may play a role in the impaired NVC observed in hAβ-KI mice. Although we observed impaired NVC under isoflurane anesthesia, isoflurane can modulate cerebral hemodynamics.^89^^–^^91^ Additional awake-mouse studies are needed to validate our NVC findings. Nonetheless, our results suggest that hAβ-KI mice exhibit NVC impairment potentially driven by astrocyte-associated OC+ granules and pericyte involvement, mirroring aspects of early human AD pathology. Future studies that investigate these cellular components may improve our understanding of impaired NVC in hAβ-KI mice.

### 4.5 Limitations

Although our findings provide important insights, several limitations should be acknowledged. First, all CBF and NVC data were performed under isoflurane anesthesia, which increases resting-state CBF levels.^89^ For NVC measurements, we lowered isoflurane to 1.2% to maximize the NVC response but maintained isoflurane at 2% for resting-state CBF measurements. This experimental design did not allow us to pool CBF measurements from mice in cohorts #1 and #3. Second, the depth penetration of LSI (∼700  μm)^92^ limits comparisons with transcriptomic and histological data, which encompass the entire cortex (∼1.2-mm thickness).^93^^,^^94^ Third, although we observed sex-specific trends, sample sizes in cohorts #2 and #3 were limited, preventing definitive sex-stratified comparisons. Finally, resting-state CBF in hAβ-KI mice was not reduced such as in traditional AD mouse models and humans, potentially due to not using old enough mice. Future studies using older mice, larger cohorts, and awake imaging protocols will be necessary to address these limitations.

## 5 Conclusions

Our study identifies specific cerebrovascular alterations in the hAβ-KI mouse model, including increased resting-state CBF, a left-shifted vessel diameter distribution, impaired NVC, and transcriptomic changes related to metabolism and inflammation. These findings not only provide insight into the cerebrovascular contributions to AD pathology but also highlight the need for further investigation into the mechanisms underlying these changes. The observed increase in resting-state CBF in the preclinical stages of the hAβ-KI model suggests a potential compensatory mechanism that may have translational relevance for early-stage AD in humans. Future studies should explore the role of sex differences in cerebrovascular dynamics, conduct NVC measurements under awake conditions, and investigate the mechanistic impact of metabolic and inflammatory gene expression changes on resting-state CBF. Expanding these studies could enhance our understanding of cerebrovascular alterations in AD and support the development of targeted therapeutic strategies.

## Supplementary Material

10.1117/1.NPh.12.S1.S14614.s01

## Data Availability

The data presented in this article is publicly available at https://doi.org/10.5061/dryad.dv41ns295
