# Supplementary material for: Cerebrovascular alterations in a mouse model of late-onset Alzheimer’s disease
Source: Neurophotonics. 2025 Jun 5;12(Suppl 1):S14614. doi: 10.1117/1.NPh.12.S1.S14614 (PMC12138534; doi:10.1117/1.NPh.12.S1.S14614)
Supplement: Supplementary file 1 [file NPh_012_S14614_SD001.pdf]

SUPPLEMENTAL METHODS

Analysis of Neurovascular Coupling Data

To minimize any potential influence of differences in resting-state CBF on NVC data, we examined the overall CBF change (ΔCBF) without normalization. The overall increase in CBF and area under the curve of CBF during stimulation were compared between WT and hAβ-KI mice. To investigate potential differences in temporal dynamics of NVC data, we calculated the $t_{50\ rise}$ and $t_{50\ fall}$. $T_{50\ rise}$ was the time after the onset of stimulation required to reach 50% of the maximum CBF before the maximum CBF response occurred due to hindpaw stimulation. $T_{50\ fall}$ was the time after the onset of stimulation required to reach 50% of the maximum CBF after the maximum CBF response occurred due to hindpaw stimulation.

Gene Set Enrichment Analysis

Pathway analysis was performed using Gene Set Enrichment Analysis (GSEA) desktop application v4.3.3 (Subramanian et al., 2005 and Mootha, Lindgren, et al. 2003). Differential expression results, generated by comparing WT and hAB-KI mice from cohort #2, were taken from DESeq2 and ranked according to the sign of the fold change ∗ -log10(adjusted p-value). All ranked genes were input into GSEA's pre-ranked analysis module. The module was set to perform 1000 permutations using the weighted enrichment statistic. The gene list was compared to MSigDB's Gene Ontology database (m5.go.v2024.1.Mm.symbols.gmt), excluding any gene sets less than 15 genes or more than 5000 genes in length. A false discovery rate < 0.25 was applied to determine significant pathways.

SUPPLEMENTAL FIGURES

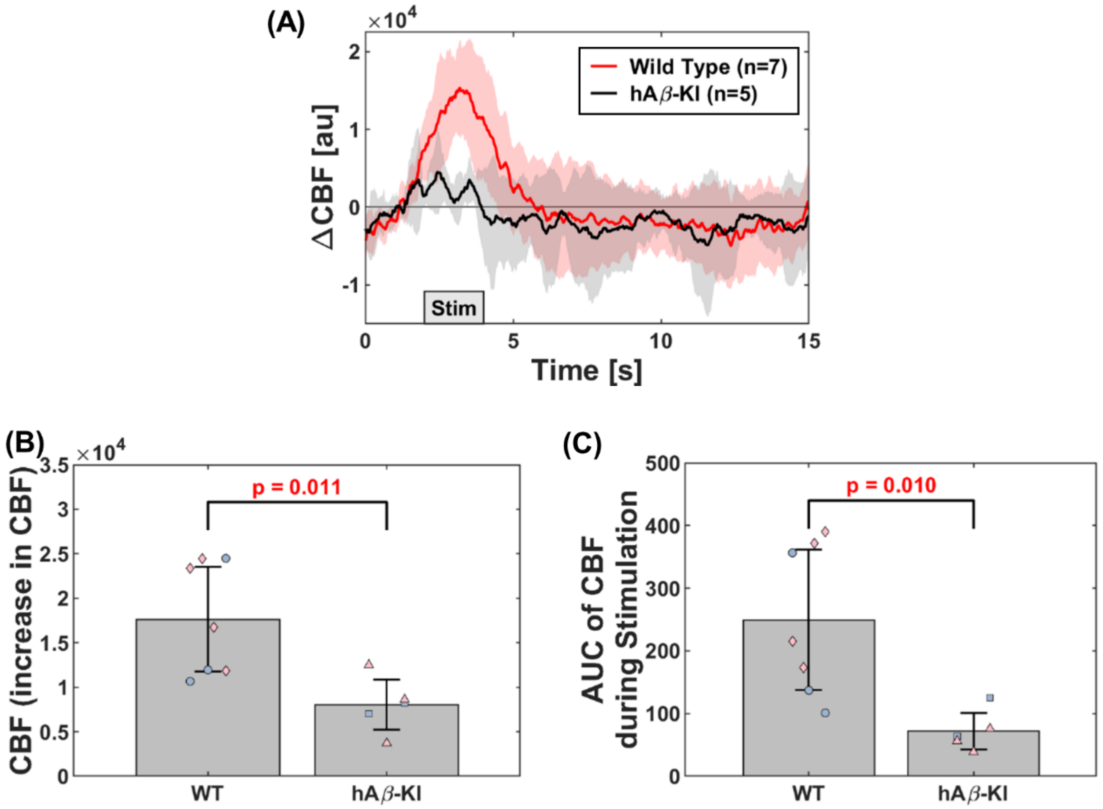

Figure S1: hAβ-KI mice have altered neurovascular coupling (NVC). (A) Change in CBF during hindpaw stimulation to assess NVC in WT (red, n = 7) and hAβ-KI (black, n = 5) mice. Shaded areas represent the standard deviation across all mice within each respective group. (B) Comparing the maximum CBF due to hindpaw stimulation between WT (blue circle = male; pink diamond = female) and hAβ-KI (blue square = male; pink triangle = female) mice (t-test). (C) Comparing the area under the curve (AUC) of ΔCBF during hindpaw stimulation between WT (blue circle = male; pink diamond = female) and hAβ-KI (blue square = male; pink triangle = female) mice (t-test). P-values are shown for each comparison, with a $p < 0.05$ considered statistically significant (red text).

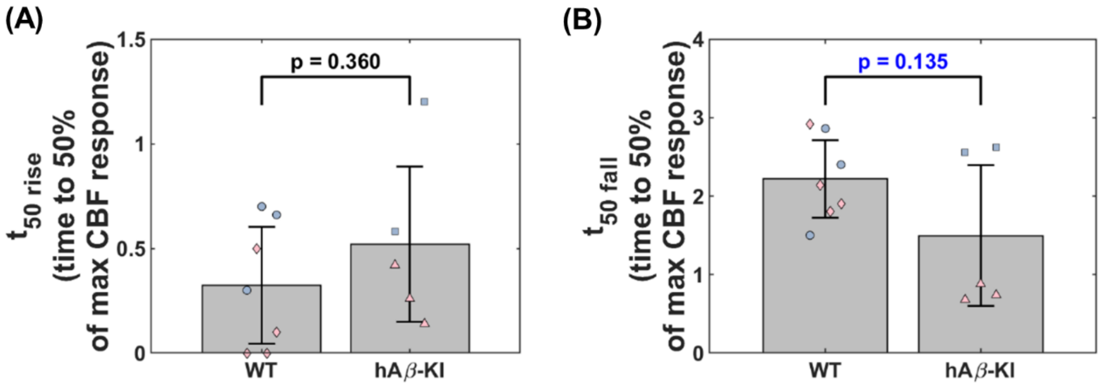

Figure S2: Temporal dynamics during neurovascular coupling (NVC) are not different in hAβ-KI mice. (A) Comparing the $t_{50\ rise}$ due to hindpaw stimulation between WT (blue circle = male; pink diamond = female) and hAβ-KI (blue square = male; pink triangle = female) mice (t-test). (B) Comparing the $t_{50\ fall}$ during hindpaw stimulation between WT (blue circle = male; pink diamond = female) and hAβ-KI (blue square = male; pink triangle = female) mice (t-test). P-values are shown for each comparison, with a $p < 0.15$ considered trending (blue text).

SUPPLEMENTAL TABLE

Table S1: Gene set enrichment analysis (GSEA) reveals downregulated pathways involved in neurotransmission.

NAME	NES	NOM p-val	FDR q-val
GOMF_NEUROTRANSMITTER_RECEPTOR_ACTIVITY	-2.569	0.000	0.047
GOCC_PRESYNAPTIC_ACTIVE_ZONE_MEMBRANE	-2.515	0.000	0.163
GOMF_POSTSYNAPTIC_NEUROTRANSMITTER_RECEPTOR_ACTIVITY	-2.497	0.003	0.180
GOMF_EXTRACELLULAR_LIGAND_GATED_MONOATOMIC_ION_CHANNEL_ACTIVITY	-2.486	0.002	0.178
GOBP_SYNAPTIC_TRANSMISSION_GABAERGIC	-2.470	0.000	0.217
